# Protective effect of *Schizonepeta tenuifolia* Briq. ethanolic extract against UVB-induced skin aging and photodamage in hairless mice

**DOI:** 10.3389/fphar.2023.1176073

**Published:** 2023-06-07

**Authors:** Min Ji Gu, Hee-Weon Lee, Guijae Yoo, Donghwan Kim, In-Wook Choi, Yoonsook Kim, Sang Keun Ha

**Affiliations:** ^1^ Korea Food Research Institute, Wanju-gun, Jeollabuk-do, Republic of Korea; ^2^ Department of Food Science and Human Nutrition, Jeonbuk National University, Jeonju-si, Jeollabuk-do, Republic of Korea; ^3^ New Drug Development Venter, Daegu-Gyeongbuk Medical Innovation Foundation, Daegu, Republic of Korea; ^4^ Division of Food Biotechnology, University of Science and Technology, Daejeon, Republic of Korea

**Keywords:** skin aging, photoaging, ultraviolet B, *Schizonepeta tenuifolia*, mitogen-activated protein kinase (MAPK), advanced glycation end products (AGEs)

## Abstract

The purpose of this study was to illuminate the mechanism by which *Schizonepeta tenuifolia* Briq. (ST) ethanolic extract prevents skin photoaging in HR-1 hairless mice (HR-1). The ST ethanolic extract alleviated wrinkle formation, epidermal skin thickness, and collagen degradation in skin tissues of ultraviolet B (UVB)-irradiated HR-1 mice. Expression of matrix metalloproteinases (a wrinkle-related marker) was reduced, and tissue inhibitor of metalloproteinase 1 expression was upregulated following application of ST ethanolic extract. Furthermore, skin dehydration and levels of hyaluronidase-1 and -2 (enzymes that break hyaluronic acid) were decreased. Moreover, protein expression of hyaluronan synthases (markers of skin hydration) and hyaluronic acid levels increased following ST ethanolic extract treatment in UVB-induced photoaging HR-1 mice. In addition, the phosphorylation of mitogen-activated protein kinases (MAPKs), including p38, extracellular signal-regulated kinase, and Jun N-terminal kinase was suppressed, and expression of nuclear factor-kappa was reduced. Treatment with ST ethanolic extract also reduced advanced glycation end product (AGE) accumulation and expression of the receptor for AGE (RAGE) in skin tissue. These results suggest that ST ethanolic extract moderates skin damage caused by UVB irradiation via regulating the expression of wrinkle- and hydration-related proteins, MAPKs, and RAGE.

## 1 Introduction

Skin photoaging includes distinct clinical, histological, and functional changes in consistently sun-exposed skin (Berneburg et al., 2000). Chronically sun-exposed skin shares features with aged skin and is caused by ultraviolet (UV) exposure, in which photoaging accelerates clinical and histological problems. Under chronic UV exposure, the skin barrier is destroyed, leading to oxidative stress, damage to the skin cells, and alteration of the protein expression of cellular signaling pathways, ultimately leading to skin pathologies such as melanoma and skin cancers, phototoxicity, and photoaging (Karin scharffetter-kochanek et al., 1997; Berneburg et al., 2000). Skin is an organ that protects the body against moisture loss, maintains body temperature, and forms a barrier against extrinsic harmful substances and physical damage, and can prevent photoaging under normal conditions. Ultraviolet B (UVB; 290–320 nm) causes pyrimidine dimer-type DNA damage and generates reactive oxygen species (ROS) in the skin, eventually leading to skin aging (Masamitsu Ichihashi et al., 2009). UVB is also closely correlated with advanced glycation end products (AGEs) accumulation in the skin (Gkogkolou and Böhm, 2015), and UV-induced oxidative stress accelerates AGEs formation, which plays a crucial role in the AGE-related modification of elastic fibers, resulting in actinic elastosis (Mizutari et al., 1997).

Skin aging is characterized as structural and functional damage caused by extrinsic and intrinsic factors, which produce changes in skin thickness, moisture dehydration, roughness, collagen degradation, and wrinkle development (Huang and Chien, 2020). These changes are closely associated with matrix metalloproteinases (MMPs), which are zinc-containing endopeptidases that degrade various constituents of the extracellular matrix (ECM) such as collagen, fibronectin, and elastin (Shin et al., 2019). The ECM proteins are involved in the skin matrix barrier, balance, and protection from extrinsic factors; however, UV induces the expression of MMP enzymes in keratinocytes and dermal fibroblasts, eventually leading to photoaging, inflammation, and cancer of the skin (Pittayapruek et al., 2016). The MMPs are ubiquitous endopeptidases; typically, MMP-1 degrades fibrillary collagen, MMP3 hydrolyzes collagens, and MMP9 digests ECM components such as collagen type I. In addition, MMPs accelerate wrinkle formation through UVB-mediated activation of nuclear factor-kappa B (NF-κB) (Berneburg et al., 2000). Photoaging and UV irradiation activate NF-κB, which upregulates MMPs, such as MMP1 and MMP3, in dermal fibroblasts (Pittayapruek et al., 2016; Shin et al., 2019).

Hyaluroic acid (HA) also known as hyaluronan, is an essential component for maintaining hydration in the epidermis and components of the extracellular matrix, and is regulated by hyaluronan synthase (HAS) and hyaluronidase (HYAL) expression. Continuous UVB exposure to the skin results in decreased HA synthesis and an attendant increase in the number of dormant fibroblasts, which are involved in cellular senescence as well as increasing the concentration of skin dehydrates, which play a major role in skin aging and exacerbate skin dehydration by activating related proteins (Averbeck et al., 2007; Sinova et al., 2022). These makers therefore need to be regulated for protection from inevitable UV irradiation and photoaging, and have been the focus of ongoing research.

*Schizonepeta tenuifolia* Briq. (ST), a member of the Labiatae family, has pharmacological properties that have been utilized by traditional herbal medicine in China, Japan, and Republic of Korea (Wang et al., 2012). The stem of ST is usually used for treatment of the common cold, headaches, fever, allergic dermatitis, pruritus, skin rashes, and eczema (Dennis Fung and Lau, 2002). Furthermore, ST has anti-oxidative, anti-inflammatory, and anti-AGE activities (Wang et al., 2012; Do et al., 2019) that involve a variety of bioactive compounds such as hesperidin, luteolin, and diosmetin (Dennis Fung and Lau, 2002; Wang et al., 2012). However, the effects of ST extract on UVB-irradiated skin photoaging have not been investigated. The aim of this study was to establish the mechanism underlying the anti-photoaging effect of ST ethanolic extract on UVB-induced skin aging in hairless (HR-1) mice. We investigated skin wrinkle- and hydration-related protein expression, and mitogen-activated protein kinases (MAPKs), NF-κB, and AGE-receptor for AGEs (RAGE) signaling pathways in UVB-irradiated mice treated with ST ethanolic extract.

## 2 Materials and methods

### 2.1 Preparation of ST ethanolic extract

The ST herb was purchased from a commercial market (Gyeongdong Market, Seoul, Republic of Korea). The sample was identified by Guijae Yoo and voucher specimen (H-166) was deposited at the Korea Food Research Institute. The sample was crushed into a powder then dissolved in 10 times its volume 30% ethanol (1:10 w/v) at 50°C for 3 h, and the process was repeated twice. The extracts were filtered, and the solvent was removed from the filtrate by rotary evaporation (R-114 Rotavapor system, Büchi Labortechnik AG, Flawil, Switzerland). Subsequently, the concentrate was lyophilized, and ST extract was stored at −20°C until use in experiments.

### 2.2 Identification of ST marker compound by high-performance liquid chromatography (HPLC)

The ST ethanolic extract was investigated with regard to its content in rosmarinic acid, used as a marker compound. Sample and reference standard solutions were prepared using 2 mg in 2 mL of 30% methanol. The HPLC was recorded under the following conditions: model–Agilent Technologies 1260 HPLC system; column: YMC Hydrosphere C18 (150 × 4.6, 5 μm); Detector: UV–Vis; mobile phase: (A) 0.1% formic acid in water with a gradient elution of 90%–10%, and (B) 0.1% formic acid in acetonitrile with a gradient elution of 10%–90%; injection volume: 10 μL; method time: 25 min; and flow rate: 1.0 mL/min; detector: UV (at 330 nm). The calibration curve was extracted using standard rosmarinic acid at concentrations between 0.125 and 0.5 μg/mL.

### 2.3 Animals

Six-week-old male HR-1 mice were purchased from Central Lab. Animal Inc. (Seoul, Republic of Korea). Experiments were conducted in accordance with the guidelines of the Ethical Committee for Animal Care and Use according to the animal protocol (approval no. IV-RA-16-2006-22). The mice were housed under consistent conditions of 23°C ± 1°C, humidity 50% ± 5%, and a 12 h light/dark cycle. After an acclimation period of 1 week, mice were randomly divided into five groups: Normal control (UVB non-irradiation control; NC), UVB irradiation control (Con), UVB + ST ethanolic extract 100 mg/kg/day (ST100), UVB + ST ethanolic extract 200 mg/kg/day (ST200), and UVB + positive control (phosphatidylserine 50 mg/kg/day; PS). All samples were diluted with distilled water and orally administered for 9 weeks. During the experimental period, food and water were provided *ad libitum*.

### 2.4 UVB-induced skin aging on HR-1 mice

The UVB was irradiated on the back skin of the mice three times a week for 9 weeks using a UV Crosslinker CL-1000S (UVP Inc., Upland, CA, United States). The energy of UVB irradiation was progressively increased from 60 mJ/cm^2^ in the first week to 120 mJ/cm^2^ in the second week, 180 mJ/cm^2^ in the third week, and 230 mJ/cm^2^ in the fourth to final weeks, using a UVB sunlamp (254 mm).

### 2.5 Serum aspartate aminotransferase (AST) and alanine aminotransferase (ALT) activity

Serum was separated from HR-1 mouse blood samples and measured using a commercial enzyme-linked immunosorbent assay (ELISA) kit to measure the activity of AST and ALT. The AST activity assay kit (MAK055, Sigma Aldrich, St. Louis, MO, United States) and ALT activity assay kit (MAK052, Sigma Aldrich) were used according to the manufacturer’s instructions.

### 2.6 Skin thickness analysis

To evaluate the skin thickness in HR-1 mice, a double layer of skin thickness was measured using a digital caliper (CD-15APX, Mitutoyo, Japan) by holding a 1-cm width below the center of the mouse back skin. Analysis was repeated thrice.

### 2.7 Skin wrinkle and moisture ration analysis

Skin wrinkle evaluation of the dorsal skin of the mice was performed at 9 weeks. Skin replicas were obtained from the dorsal skin surface of HR-1 mice using a replica kit (Epigem Inc., Seoul, Republic of Korea) and analyzed to measure skin wrinkles (Visioline VL650, CK Electronics GmbH, Cologne, Germany). The assessment of skin wrinkles was the srinkle area, total wrinkle length, and mean wrinkle depth. To evaluate the skin moisture rate, the skin moisture content was measured on the back skin of HR-1 mice using a skin analysis device (WillCam, K. L. Global, Seoul, Republic of Korea).

### 2.8 Skin histological analysis

To identify histological alterations, dorsal skin was obtained and fixed in 10% formalin. The fixed skins were embedded in paraffin and sliced into 4-μm sections. Sliced sections were stained with hematoxylin and eosin (H&E) for skin epidermis thickness analysis and Masson’s trichrome stain for collagen fiber analysis. To measure the expression of AGEs, immunohistochemistry (IHC) was performed on skin tissue. Images were obtained using a digital slide scanner (Motic Easyscan one, Motic, Hong Kong), and skin epidermis thickness was measured using image analysis software (Motic DS Assistant, Hong Kong).

### 2.9 Western blotting

Mouse back skin tissues were lysed using radioimmunoprecipitation assay buffer (Cell Signaling, Beverly, MA, United States) containing phosphatase and protease inhibitors (Sigma-Aldrich). Proteins were quantified using a detergent compatible protein assay kit (Bio-Rad, Hercules, CA, United States). The proteins were separated by 8%–12% sodium dodecyl sulfate-polyacrylamide gel electrophoresis and electro-transferred onto an immunoblot nitrocellulose membrane (Bio-Rad, Montreal, Canada). The membranes were blocked with 5% skim milk in Tris-buffered saline containing 0.2% Tween-20 (TBST) for 1 h at 23°C ± 2°C. After blocking, the membranes were incubated with the primary antibodies overnight at 4°C and washed with TBST. The membranes were then incubated with secondary antibodies at 23°C ± 2°C for 1 h. After incubation, proteins were visualized using the enhanced detection reagent Lumi Femto solution (Dogen Bio, Seoul, Republic of Korea) and imaged using the ChemiDoc XRS^+^ imaging system (Bio-Rad) and Image Lab software (version 4.1, Bio-Rad). Each protein was quantitated using ImageJ software (National Institutes of Health, Bethesda, MD, United States), and β-actin was used as an internal control for densitometry.

### 2.10 Tissue inhibitor of metalloproteinase 1 (TIMP1), HYAL1, HYAL2, and HA quantification analysis

To measure the TIMP1 in mouse skin tissues, we used a TIMP1 ELISA kit (ab196265; Abcam, Cambridge, UK). Hyaluronidase-1 ELISA (MBS9395469, MYBiosource, San Diego, CA, United States) and hyaluronidase-2 ELISA kits (MBS28865; MYBiosource) were used to measure the levels of HYAL1 and HYAL2 expression in mouse skin tissue. A hyaluronan quantikine ELISA kit (DHYAL0; R&D systems, Minneapolis, MN, United States) was used for quantitative analysis of hyaluronan hyaluronic acid in mouse back skin tissue. All ELISA kits were used according to the manufacturer’s instructions.

### 2.11 Statistical analysis

Results are expressed as mean ± standard error of the mean (SEM). Statistical analysis was performed using the Graphpad Prism 7.0 software (GraphPad Software, La Jolla, CA, United States). One-way analysis of variance (ANOVA) was used for statistical analysis, and Dunnett’s *post hoc* test was performed with a significance value of *p* < 0.05.

## 3 Results

### 3.1 HPLC analysis of ST ethanolic extract quality

**FIGURE 1:**
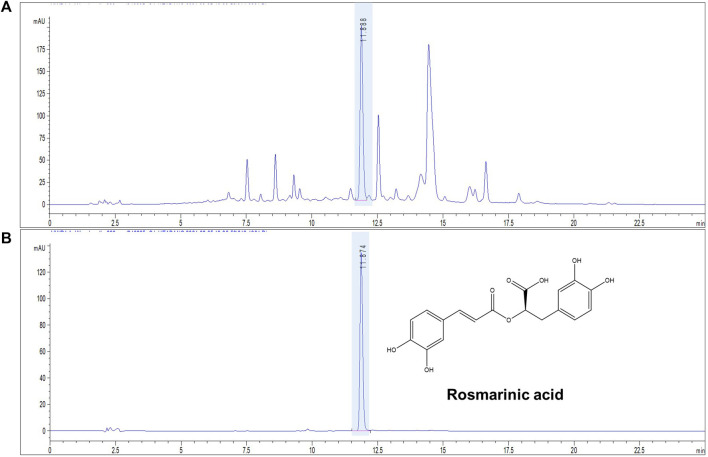
Representative chromatograms of ST ethanolic extract. Chromatogram of ST using a high-performance liquid chromatography (HPLC) chemical marker profile. HPLC-ultraviolet chromatogram; absorbance: 330 nm. **(A)** ST ethanolic extract and **(B)** rosmarinic acid standard.

The concentration of marker compound contained in ST ethanolic extract was quantified using HPLC analysis. The identification results are shown in Figure 1. Rosmarinic acid was the most abundant compound in the ST ethanolic extract. The concentration of marker in the ST ethanolic extract was 0.4321%. The proposed HPLC method was validated by determining the linearity, limit of detection and quantification, inter- and intra-day precision, repeatability, stability, and recovery; the results are summarized in Tables 1, 2. These results demonstrate that the developed HPLC method is suitable for analyzing the constituents of ST ethanolic extract.

**TABLE 1:** High-performance liquid chromatography method validation of linearity range, limit of detection, and limit of quantification using rosmarinic acid analyte.

Regression equation	Correlation coefficient (*R* ^2^)	Linear range (μg/μL)	LOD^a^ (μg)	LOQ^b^ (μg)
*y* = 2,652.0865x + 2.6055	0.9993	0.03125–0.5000	0.0239	0.0725

^a^ Limit of detection.

^b^ Limit of quantification.

**TABLE 2:** High-performance liquid chromatography evaluation of precision, stability, and accuracy (recovery) using rosmarinic acid analyte.

Precision RSD^a^ (%)							
Analyte concentration (μg/mL)		Intra-day (*n* = 3)		Inter-day (*n* = 3)		Repeat-ability RSD (%) (*n* = 5)	
0.500		0.063		0.088		1.148	
0.250		0.171		0.174			
0.125		0.393		0.108			
Accuracy							
Sam:Std	Spiked (μg)		Detected (μg)		Recovery (%)		RSD (%)
1:2	1.811		1.837		101.470		1.235
1:1	1.466		1.572		107.227		0.146
2:1	1.121		1.106		98.650		2.826

^a^ Relative standard deviation; Sam, Sample; Std; Standard.

### 3.2 Effect of ST ethanolic extract on body weight and serum biochemical markers

**FIGURE 2:**
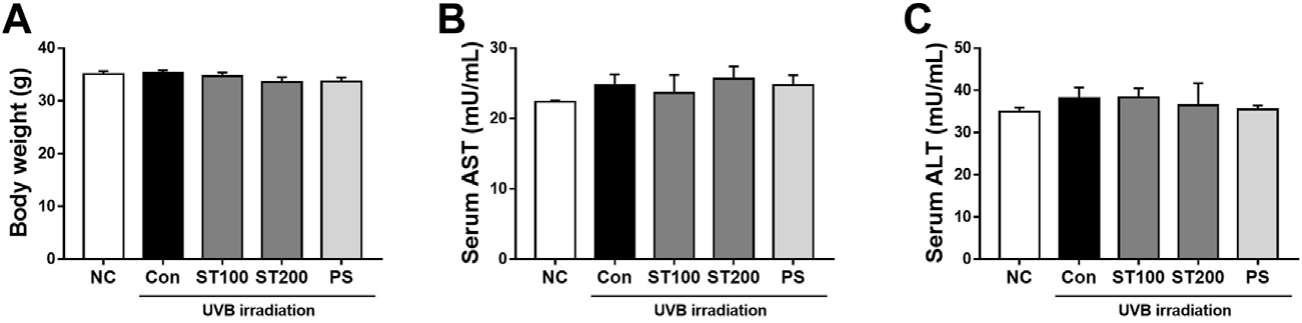
Change of body weight and liver toxicity serum biomarkers in hairless HR-1 mice following ultraviolet (UV) B-induced skin photoaging with ST ethanolic extract treatment. **(A)** Body weight change in the final week. **(B)** Change of the serum aspartate aminotransferase (AST) levels and **(C)** alanine aminotransferase (ALT) levels. Results are presented as the mean ± SEM (*n* = 8).

To evaluate changes in the body weight of the HR-1 mice, their body weight was measured at 9 weeks. Body weight did not differ significantly between the groups (Figure 2A). To determine the toxicity of the ST ethanolic extract, we examined serum ALT and AST levels using an ELISA kit. As shown in Figures 2B, C, serum AST and ALT levels were not significantly altered in any of the groups of HR-1 mice. These data suggest that body weight, serum AST, and serum ALT levels were not altered by oral administration of ST ethanolic extract.

### 3.3 Effect of ST ethanolic extract on skin wrinkle formation by UVB-induced photoaging

**FIGURE 3:**
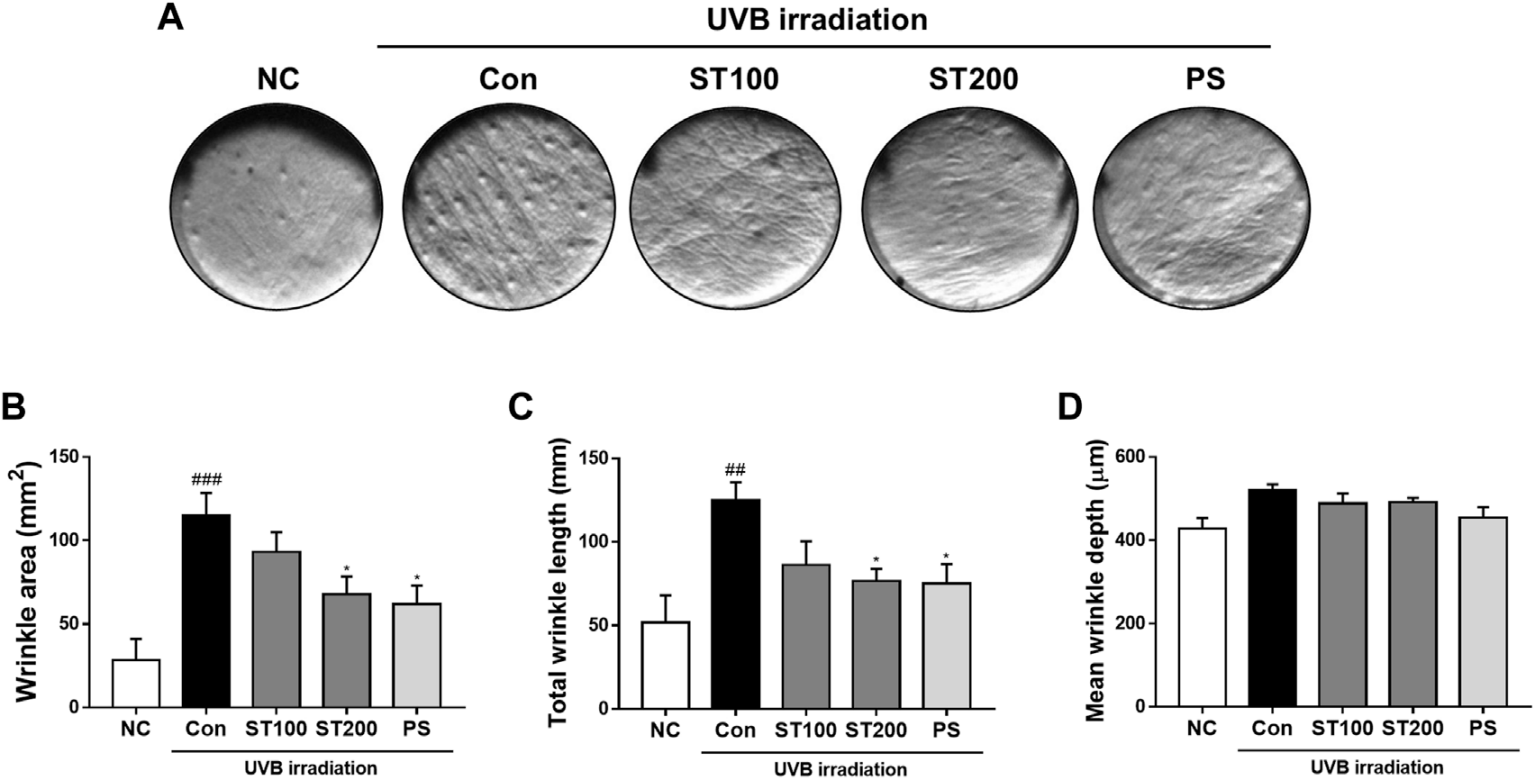
Effects of ST ethanolic extract administration on ultraviolet (UV)B-induced formation of wrinkles in the skin tissue of hairless HR-1 mice. **(A)** Representative histological results of replica analysis. **(B)** Wrinkle area, **(C)** total wrinkle length, and **(D)** mean wrinkle depth. Results are presented as the mean ± SEM (*n* = 3). ^#^ *p* < 0.05, ^##^ *p* < 0.01, and ^###^ *p* < 0.001 vs. normal control; NC, *p < 0.05, **p < 0.01, and ***p < 0.001 vs. UVB irradiation control; Con.

To examine the effect of the ST ethanolic extract on wrinkle formation in HR-1 mice, skin replica analysis was performed on the back skin. Wrinkle formation was markedly increased in the UVB irradiation group compared to that in the normal control group; however, after oral administration of ST ethanolic extract, skin wrinkles tended to decrease compared with that in the UVB control group (Figure 3A). As shown in Figures 3B, C, wrinkle area and total wrinkle length were reduced by 40.9% and 38.8%, respectively in the ST ethanolic extract (200 mg/kg) treatment group compared with that in UVB control group. The mean wrinkle depth tended to decrease following ST ethanolic extract administration (Figure 3D). These results indicate that wrinkle formation was reduced by ST ethanolic extract treatment during UVB-induced photoaging.

### 3.4 Inhibitory effect of ST ethanolic extract on epidermal thickness and collagen degradation by UVB-induced photoaging

**FIGURE 4:**
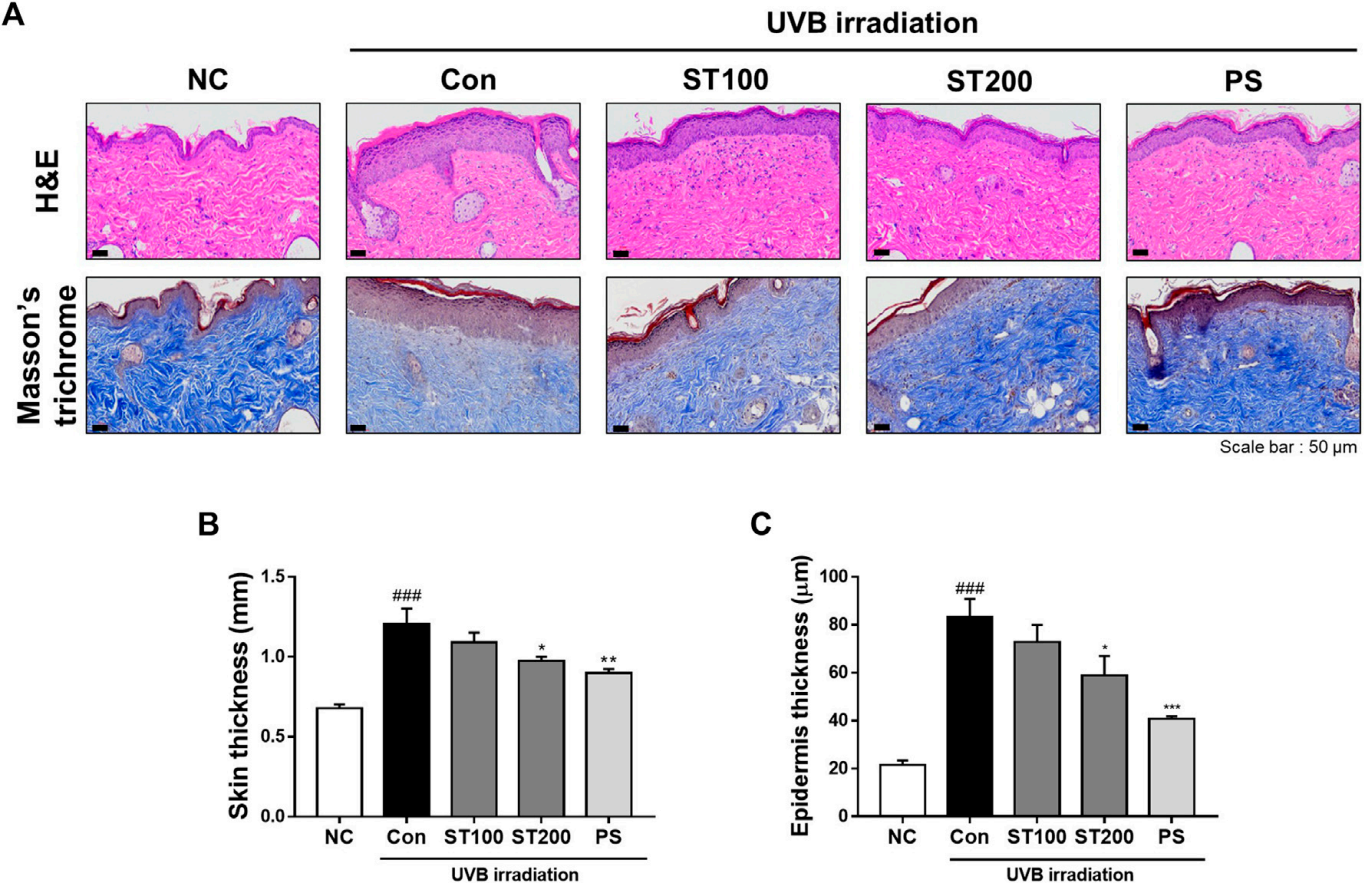
Effects of ST ethanolic extract administration on ultraviolet (UV)B-induced skin thickness and collagen degradation in skin tissue of hairless HR-1 mice. **(A)** The skin thickness was observed by hematoxylin and eosin (H&E) staining. Masson’s trichrome stain was used to identify degradation of skin fibers. Representative microscope images were taken from three independent experiments; Scale bar = 50 μm. **(B)** The bar graph shows the percentage of epidermal thickness (H&E staining data) relative to the normal control, measured using ImageJ software. **(C)** The skin thickness was determined using skinfold thickness analysis. Results are presented as the mean ± SEM (*n* = 3). ^#^ *p* < 0.05, ^##^ *p* < 0.01, and ^###^ *p* < 0.001 vs. normal control; NC, *p < 0.05, **p < 0.01, and ***p < 0.001 vs. UVB irradiation control; Con.

To estimate the effects of ST ethanolic extract on UVB-induced skin thickness and collagen degradation, dorsal skin sections of HR-1 mice were stained with H&E and Masson’s trichrome, respectively. After consecutive UVB irradiation, epidermal thickness was extended in the UVB control group compared with that in the normal control group; however, skin thickness was reduced following oral administration of ST ethanolic extract compared with that of the UVB control group (Figure 4A). In addition, we observed that the UVB-induced reduction in collagen fibers was recovered after oral administration of ST ethanolic extract. As shown in Figure 4B, after oral administration of ST ethanolic extract (100 and 200 mg/kg), the skin thickness was reduced by 9.5% and 19.1%, respectively, compared with that in the UVB control group. In addition, the epidermis thickness was decreased following treatment with ST ethanolic extract (100 and 200 mg/kg) by 12.8% and 29.5%, respectively, compared with that in the UVB control group; the ST ethanolic extract 200 mg/kg group showed especially significant differences (Figure 4C). These results suggest that ST ethanolic extract may improve skin thickness and collagen density during UVB-induced photoaging.

### 3.5 Effect of ST ethanolic extract on skin wrinkle-regulation factors following UVB-induced photoaging

**FIGURE 5:**
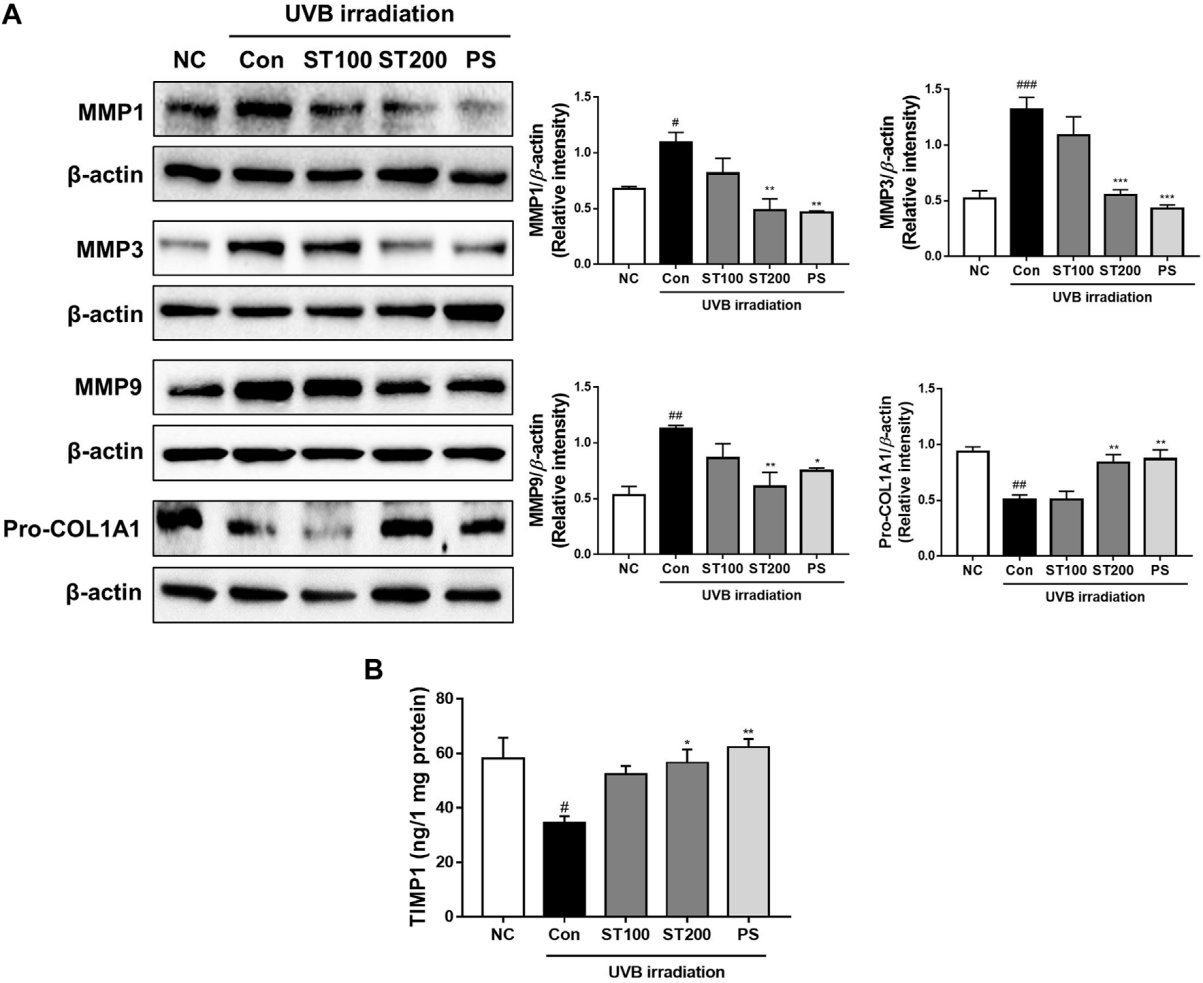
Effect of ST ethanolic extract administration on ultraviolet (UV)B-induced expression of matrix metalloproteinases (MMPs), pro-collagen, and tissue inhibitor of metalloproteinase 1 (TIMP1) following UVB-irradiation of skin tissue in hairless HR-1 mice. HR-1 mice were orally administered 100 or 200 mg/kg of ST ethanolic extract for 9 weeks. **(A)** Expression of MMPs (MMP1, 3, and 9) and pro-collagen A1 was detected by Western blot analysis and quantitative values of protein expression in HR-1 mice. All results were normalized to the normal control and calculated using ImageJ software. **(B)** The level of expression of TIMP1 was detected using an ELISA kit. Results are presented as the mean ± SEM from three independent experiments. ^#^ *p* < 0.05, ^##^ *p* < 0.01, and ^###^ *p* < 0.001 vs. normal control; NC, *p < 0.05, **p < 0.01, and ***p < 0.001 vs. UVB irradiation control; Con.

We examined whether ST ethanolic extract inhibited the expression of MMP 1, 3, and 9 at the protein level in UVB-irradiated HR-1 mice using Western blotting. As shown in Figure 5A, UVB treatment increased the expression of MMP1, 3, and 9 by 161.5%, 252.2%, and 211.9%, respectively; however, oral administration of ST ethanolic extract significantly reduced these protein levels in the 200 mg/kg treatment group by 55.4%, 58.2%, and 46%, respectively. In addition, UVB exposure significantly upregulated the expression of pro-collagen A1 in the 200 mg/kg ST ethanolic extract treatment group. Furthermore, the level of TIMP1, a tissue inhibitor of metalloproteinases, was downregulated by UVB irradiation; however, oral administration of 200 mg/kg ST ethanolic extract upregulated the level of TIMP1 by 163.6% compared with that in the UVB control group (Figure 5B). These data suggest that ST ethanolic extract may regulate MMPs and TIMP1 expression in HR-1 mice exposed to UVB irradiation.

### 3.6 Effect of ST ethanolic extract on skin hydration-regulation factors following UVB-induced photoaging

**FIGURE 6:**
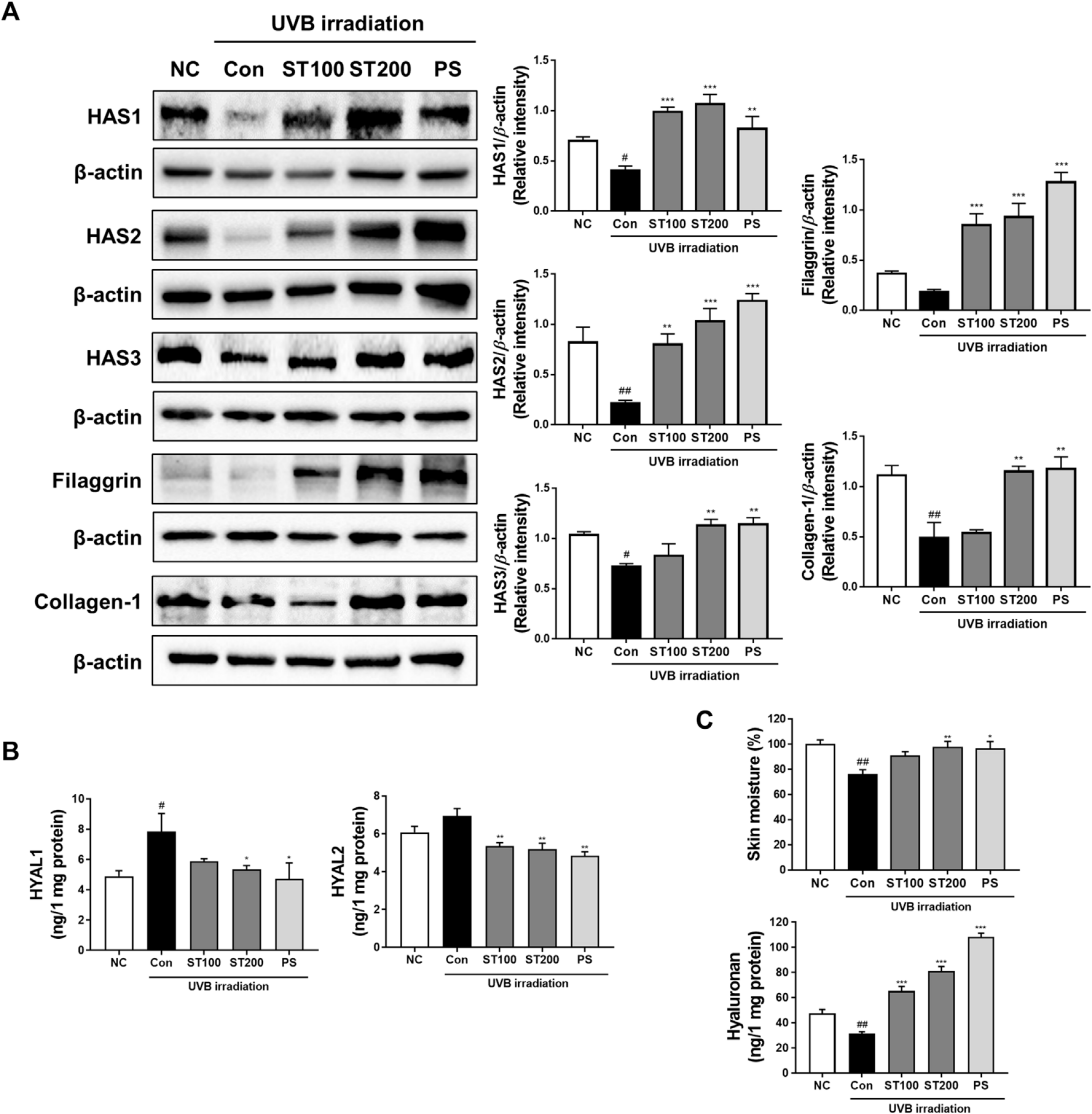
Effect of ST ethanolic extract administration on ultraviolet (UV)B-induced expression of hydration regulating proteins in skin tissue of HR-1 mice. HR-1 mice were orally administered 100 or 200 mg/kg of ST ethanolic extract for 9 weeks. **(A)** Expression of the hyaluronan synthase (HAS) family (HAS1, 2, and 3), filaggrin, and collagen A1 was detected using Western blot analysis and quantitative values of protein expression in HR-1 mice. All results were normalized to the normal control and calculated using ImageJ software. **(B)** Expression levels of hyaluronidase (HYAL)1 and 2. **(C)** Dosal skin moisture (%) and hyaluronan expression levels were detected using an ELISA kit. Results are presented as the mean ± SEM from three independent experiments. ^#^ *p* < 0.05, ^##^ *p* < 0.01, and ^###^ *p* < 0.001 vs. normal control; NC, *p < 0.05, **p < 0.01, and ***p < 0.001 vs. UVB irradiation control; Con.

To determine whether ST ethanolic extract enhanced skin hydration following UVB-induced skin damage, we performed Western blot analysis in HR-1 mice. As shown in Figure 6A, UVB irradiation downregulated the expression of HAS enzymes (HAS 1, 2, and 3), and protein levels were significantly decreased compared with that in the normal control group. Administration of ST ethanolic extract (100 and 200 mg/kg) increased the expression of HAS 1, 2, and 3 compared to that in the UVB irradiation group. Expression of filaggrin, which protects the skin barrier and maintains skin hydration (Mildner et al., 2010), was increased in the 100 mg/kg and 200 mg/kg ST ethanolic extract treatment groups by 4.6- and 5.0-fold, respectively, compared with that in the UVB control group. Moreover, our results showed that HYAL1 and 2 increased protein levels in the UVB irradiation group, but the ST ethanolic extract treatment groups showed significantly decreased levels compared with that in the UVB control group (Figure 6B). Additionally, as shown in Figure 6C, skin moisture and hyaluronan levels were upregulated by ST ethanolic extract treatment. These results indicate that ST ethanolic extract may enhance skin hydration by upregulating HAS enzymes and filaggrin, concurrently downregulating HYALs in HR-1 mice exposed to UVB irradiation.

### 3.7 Effect of ST ethanolic extract on MAPKs and NF-κB expression following UVB-induced photoaging

**FIGURE 7:**
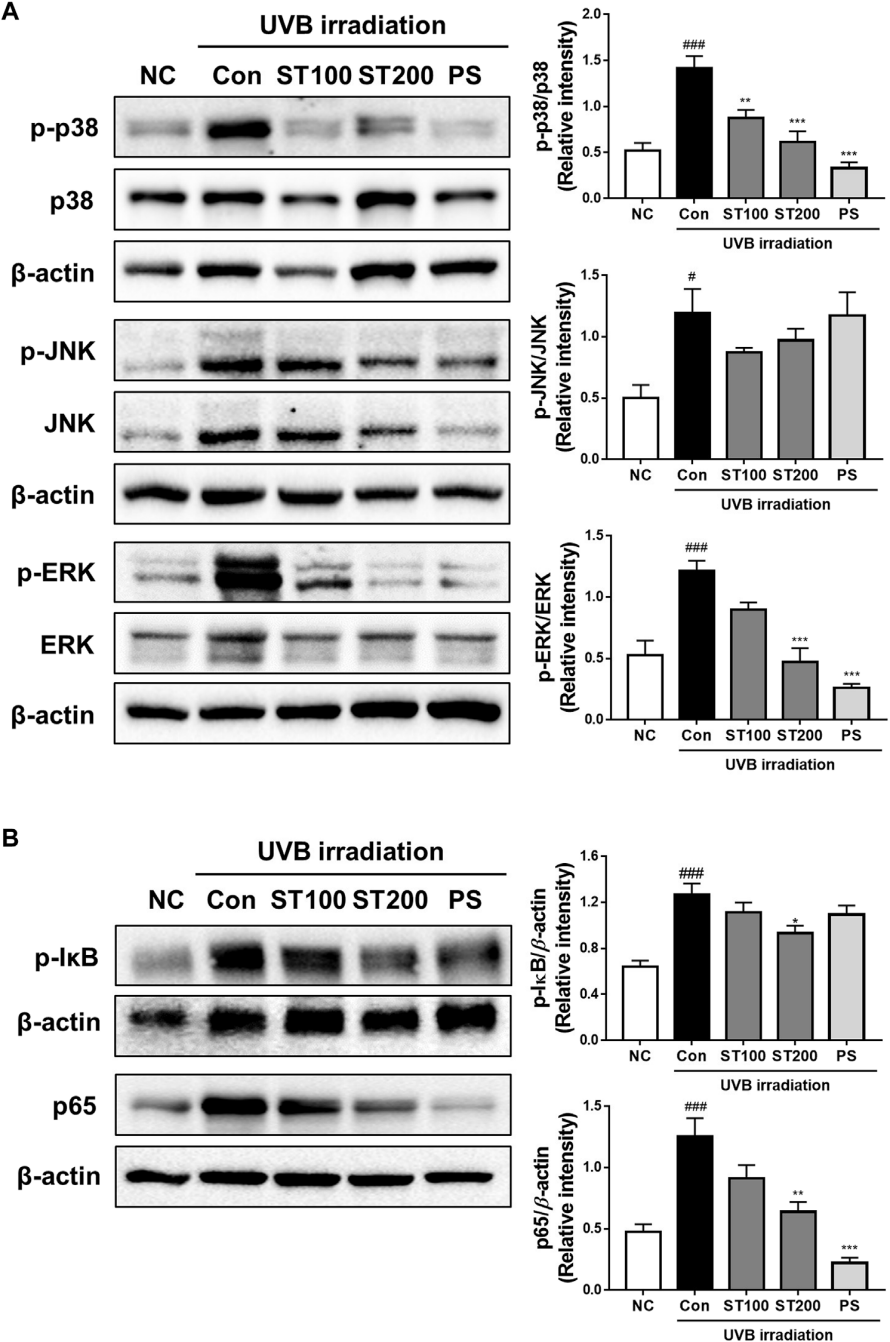
Effect of ST extract administration on ultraviolet (UV)B-induced expression of mitogen-activated protein kinases (MAPKs) and nuclear factor-kappa B (NF-κB) in skin tissue of hairless HR-1 mice. HR-1 mice were orally administered 100 or 200 mg/kg ST ethanolic extract for 9 weeks. **(A)** The expression of phosphorylated p-38 (p-p38), Jun N-terminal kinase (p-JNK), and extracellular signal-regulated kinase (p-ERK) was detected using Western blot analysis and quantitative values of MAPKs expression in HR-1 mice. **(B)** Expression of phosphorylation IκB and NF-κB (p65) in HR-1 mice and quantitative values of phosphorylation IκB and NF-κB (p65) expression in HR-1 mice. All results were normalized to the normal control and calculated using ImageJ software. Results are presented as the mean ± SEM from three independent experiments. ^#^ *p* < 0.05, ^##^ *p* < 0.01, and ^###^ *p* < 0.001 vs. normal control; NC, *p < 0.05, **p < 0.01, and ***p < 0.001 vs. UVB irradiation control; Con.

We examined the effect of UVB irradiation on the activation of MAPKs and the NF-κB signaling pathway. As shown in Figure 7A, UVB irradiation induced the phosphorylation of p38, Jun N-terminal kinase (JNK), and extracellular signal-regulated kinase (ERK) by 2.7-, 2.3-, and 2.4-fold, respectively, compared with that in normal controls. Treatment with ST ethanolic extract suppressed the expression of phosphorylated p38, JNK, and ERK compared with that in the UVB irradiation group. In addition, the phosphorylation of IκB increased in UVB control group; however, ST treated group changed the phosphorylation levels, significantly in the ST200 group. The activation of NF-κB in the UVB control group was higher than that in the normal control group; however, the ST ethanolic extract group showed a significant decrease in the ST200 group (Figure 7B). These data suggest that ST ethanolic extract prevents UVB-induced skin damage by downregulating the activation of MAPKs and the NF-κB signaling pathway.

### 3.8 Effect of ST on AGE and RAGE expression following UVB-induced photoaging

**FIGURE 8:**
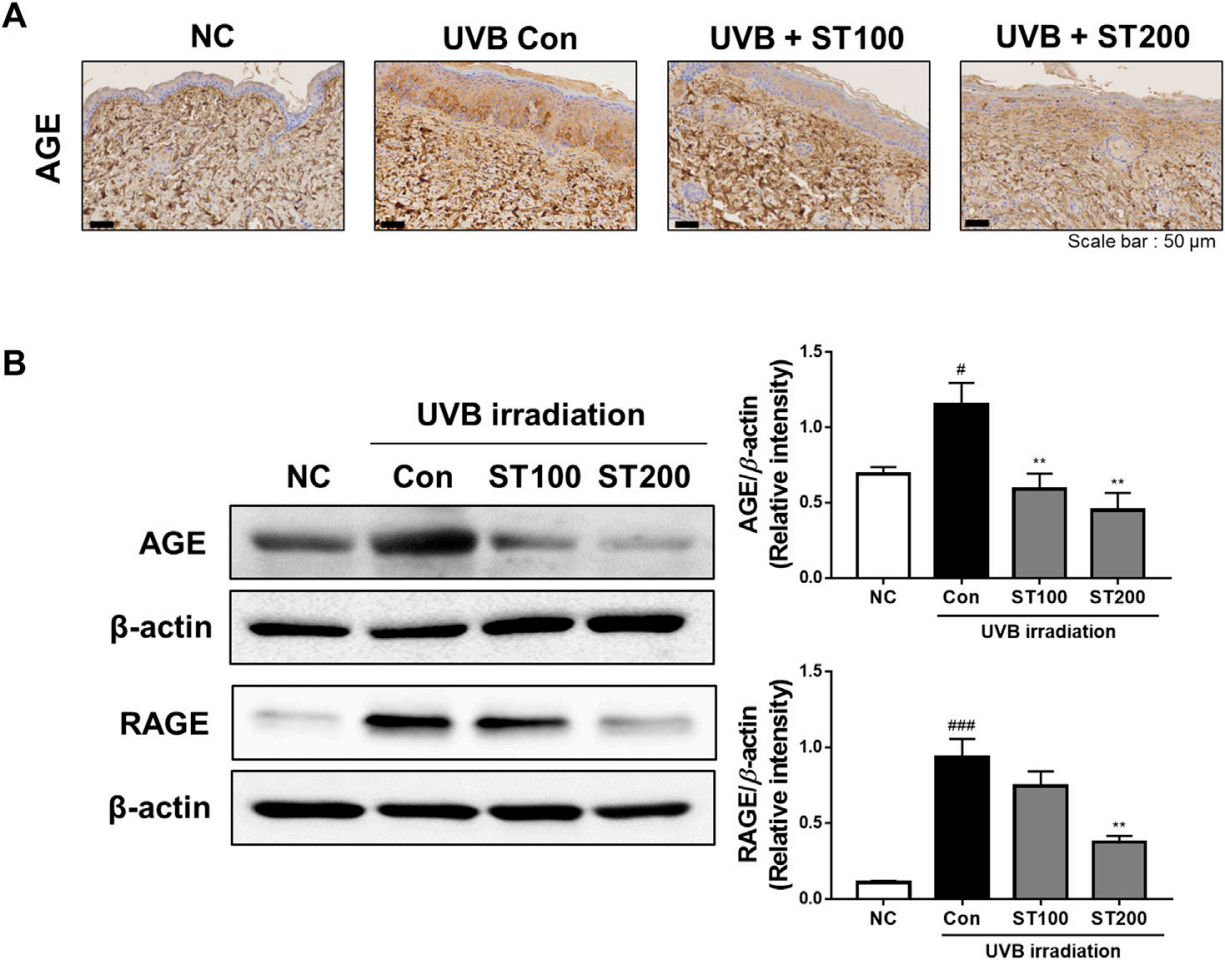
Effect of ST ethanolic extract administration on ultraviolet (UV)B-induced expression of advanced glycation end products (AGEs) and receptor for AGEs (RAGE) in skin tissue of hairless HR-1 mice. HR-1 mice were orally administered 100 or 200 mg/kg of ST ethanolic extract for 9 weeks. **(A)** Immunohistochemistry analysis of AGEs accumulation in the skin of HR-1 mice. Representative microscopic images were obtained from three independent experiments; scale bar = 50 μm. **(B)** Expression of AGEs and RAGE was detected using Western blot analysis and quantitative values of the expression of AGEs and RAGE in HR-1 mice. All results were normalized to the normal control and calculated using ImageJ software. Results are presented as the mean ± SEM from three independent experiments. ^#^ *p* < 0.05, ^##^ *p* < 0.01, and ^###^ *p* < 0.001 vs. normal control; NC, *p < 0.05, **p < 0.01, and ***p < 0.001 vs. UVB irradiation control; Con.

In the present study, we observed the expression of AGEs and RAGE using IHC analysis and found that UVB irradiation accelerated AGE accumulation and RAGE-expression upregulation. As shown in Figure 8A, UVB irradiation increased expression of AGEs in the UVB control group compared to that in the normal control group. Treatment with 200 mg/kg ST ethanolic extract markedly reduced the accumulation of AGEs. To further elucidate the regulation of AGEs and RAGE by ST ethanolic extract, we determined protein expression using Western blot analysis. As shown in Figure 8B, ST ethanolic extract inhibited the accumulation of AGEs and upregulation of RAGE. These results demonstrate that ST ethanolic extract inhibited the expression of AGEs and RAGE following UVB-induced AGE accumulation in HR-1 mice.

## 4 Discussion

Skin aging is a natural process that prolongs human life, but it is accelerated by a combination of external factors, such as UV radiation, nutritional conditions, and chemical pollution (Cao et al., 2020). Among the skin aging factors, photoaging is the most critical cause of skin damage and aging. Diverse foodborne agents have been investigated for their potential protective effects for the body and skin (Geng et al., 2021). In this study, UVB irradiation induced skin photoaging, which was protected against by the regulation of MMPs, HAS, and MAPK pathways in HR-1 mice treated with ST ethanolic extract. In addition, the ST ethanolic extract regulated the AGE-RAGE pathway by inhibiting AGE accumulation in the skin.

ST is contains glucosides, volatile oils, and flavonoids such as hesperetin and luteolin, those phytochemicals have reported as natural inhibitors against oxidative stress and inflammation (Dennis Fung and Lau, 2002). We quantified the rosmarinic acid from ST ethanolic extract using HPLC. As shown in Figure 1, the rosmarinic acid is most abundant phytochemical in the ST ethanolic extract, also, it has reported in other studies to be found in ST (Lee et al., 2008; Kang et al., 2010). Rosmarinic acid is a polyphenol as a flavonoid family, which has phenolic carboxylic acid and methyl ester structure (Juana et al., 2012; Zhao and Zhou, 2022). Flavonoids are known to have therapeutic effects, including anticancer, antimicrobial, antioxidant, and antitumor, these effects came from the chemical structure properties of unsaturation and oxidation of carbon ring (Ullah et al., 2020). In this context, rosmarinic acid has reported as a free radical scavenger against UV irradiation, which photoprotection effect have demonstrated through the decrease of UV-induced oxidative stress *in vitro* model (Sanchez-Campillo et al., 2009). Sanchez-Campillo et al. suggested that rosmarinic acid may be beneficial as functional, nutraceutical and pharmaceutical product for oral administration and dermatologic photo-protective formulations. It has been reported that rosmarinic acid exhibits the effect of anti-photodamage from UVB exposure in chronic animal model (Balb/C mice) by prevention endoplasmic reticulum stress and impairment of mitochondrial dynamics via alleviation of the oxidative stress (Gupta et al., 2023). Therefore, we expected that ST ethanolic extract containing rosmarinic acid would have potential effect on UVB-induced skin photodamage. In this study, we investigated the mechanism underlying the effects of ST esthanolic extract on skin damage induced by UVB-irradiation in HR-1 mice. As a result, the ST might be used as a functional food ingredient to modulate the mechanisms involved in skin photodamages.

Constantly exposing the skin to UV damage causes complex phenotype changes of skin tissue. UVB irradiation induces skin aging, which increases wrinkles, rough skin texture, fragility, and dryness (Chung et al., 2003). In this study, we observed oral administration of ST ethanolic extract alleviated skin damage by reducing skin thickness, collagen degradation, and wrinkle formation in HR-1 mice following UVB-induced photoaging. Skin aging by UVB irradiation regulates wrinkle- and hydration-regulated enzymes, such as MMPs and the HAS family (Pittayapruek et al., 2016; Garg, 2017; Sinova et al., 2022). We showed that irradiation of HR-1 mice increased MMP1, MMP3, and MMP9 expression, which decreased following ST ethanolic extract administration. In turn, expression of pro-collagen A1 and TIMP1 levels increased. These results were also consistent with reported studies, suppression of MMPs by TIMP1 protects and recovers ECM degradation and inflammation following UV irradiation (Kähäri and Saarialho-Kere, 1997). Yokose et al. demonstrated that TIMP1 expression is decreased in aged skin xenografted onto severely immune-deficient mice (Yokose et al., 2012). Another study reported that most skin cells, such as keratinocytes, fibroblasts, and melanocytes, produce several classes of TIMPs, and the activity of MMPs is determined by the balance of MMPs to TIMPs (Pillai et al., 2005). In our study, the level of TIMP1 increased following ST ethanolic extract administration and was significantly altered at a concentration of 200 mg/kg. In addition, phosphatidayserine (PS), as used positive control in our study, reported that the prevention of skin aged, which increases procollagen synthesis and inhibits MMP-1 expression in UV-irradiation human skin (Cho et al., 2008). In another study, polyphenolic-rich ethanolic *Spatholobus suberectus* stem extract protected against ROS and cellular damage via inhibition of MMPs, upregulation of TIMP1, and the blockade of MAPK phosphorylation following UVB-induced skin photoaging in human epidermal keratinocytes (HaCaT) (Kwon et al., 2019). In addition, polyphenols extracted from Kuding tea have antioxidant effects and inhibit UVB-induced skin degradation through the downregulation of MMPs, upregulation of TIMPs, and activation of anti-oxidative enzymes (Yi et al., 2019). Therefore, the activation of MMPs and TIMPs plays an important role in collagen production, and ECM degradation consistently leads to skin aging. As expected, in this study, the damage caused by UVB radiation was reduced following ST ethanolic extract administration. These results imply that the ST ethanolic extract alleviates UVB-induced photoaging by regulating the expression of MMPs and TIMPs.

Skin dehydration is a crucial component of skin aging, and the levels of hydration are reduced upon UVB exposure. Skin hydration is retained by molecules such as HA, glycosaminoglycan, and water molecules (Papakonstantinou et al., 2012). Water that is HA-bound in the dermis and epidermis is involved in skin hydration, which plays an important role in collagen and ECM structural maintenance (Papakonstantinou et al., 2012; Kwon et al., 2019). The degradation of skin collagen and ECM structure reduces skin elasticity, which alters the thickness and produces wrinkles in the skin. A previous study reported that HA is synthesized by HAS in HAS-knockdown human fibroblasts, while it is destroyed by HYAL in mouse back skin under chronic UVB irradiation (Dai et al., 2007). In addition, filaggrin is known to protect the epidermal barrier of the skin from dryness, which retains the hydration of the epidermis through involvement of skin moisturizing factor (McGrath and Uitto, 2008). In our study, HA decreased in the skin of UVB-induced photoaging mice; however, it was significantly increased in the ST ethanolic extract-treated group. The ST ethanolic extract treatment groups showed notably recovered expression of HAS 1, 2, and 3, while the expression of HYAL 1 and 2 was downregulated. This result indicated the regulation of HAS and HYAL enzymes by ST ethanolic extract treatment in mice exposed to UVB irradiation. Furthermore, filaggrin expression decreased upon UVB irradiation, and as expected, was restored by treatment with 200 mg/kg of ST ethanolic extract. These results are in agreement with another study that showed HA was enhanced via regulation of HAS1 and HYAL1 and modulated by filaggrin in HaCaT cells treated with *Aloe vera* flower water extract (Razia et al., 2021). In another study, skin hydration factors including filaggrin and HA levels were upregulated, and HYAL levels were downregulated by an active compound of *Hydrangea serrata* extracts in a UVB-induced photoaging HR-1 mice, which prevented wrinkle formation and skin dehydration (Myung et al., 2019). The present study showed that ST ethanolic extract increased HA levels by enhancing HAS enzymes and filaggrin levels, and in turn, reduced the expression of HYAL enzymes in UVB-irradiated HR-1 mice. These results suggest that ST ethanolic extract may enhance skin hydration, leading to anti-photoaging effects in the skin.

Ultraviolet-induced ROS upregulate transcription factors, including MAPKs, which play a role in triggering various inflammatory disorders in the skin (Katiyar et al., 2001). Chronic irradiation with UVB activates the p38 MAPK signal transduction pathways and NF-κB, which induce a pro-inflammatory response in the skin of HR-1 mice (Kim et al., 2005). In addition, the expression of activating protein-1 (AP-1) and NF-κB was induced in human skin and MMPs, which leads to the degradation of matrix proteins by UVB exposure (Berneburg et al., 2000). In addition, NF-κB is activated by UV irradiation and correlates with MMP-1 levels in skin keratinocytes; therefore, UVB-induced skin photoaging may be inhibited by blocking the NF-κB pathway (Tanaka et al., 2010; Kwon et al., 2019). In addition, PS reported the inhibit of MMP-1 by downregulation of MAPKs in human dermal fibroblast cell line (Lee et al., 2013). Recently, these signaling pathways have been targeted to protect against UV-induced skin photodamage through treatment with natural substances. In this regard, a recent study reported that during UVB irradiation in HR-1 mice, skin damage, including oxidative stress, inflammatory responses, and DNA damage, were protected against by blackberry extract administration via modulated MAPKs and NF-κB (Divya et al., 2015). In another study, the expression of MAPKs, NF-κB, and AP-1 signaling was inducted in UVB-irradiated HR-1 mice, which was suppressed by treatment with hawthorn polyphenol extract, leading to reduced skin photoaging through inhibition of oxidative stress and MMP production (Liu et al., 2018). In our study, we showed that ST ethanolic extract suppressed UVB-induced phosphorylation of IκB, its lead to activation of NF-κB and phosphorylation of MAPKs in the skin of HR-1 mice. These results suggest that the ST ethanolic extract attenuates UVB-induced photoaging by blocking the phosphorylation of MAPKs and inactivating NF-κB in the skin of HR-1 mice, thereby regulating MMP expression.

Skin photoaging is exacerbated by UV exposure, which leads to increased glycation stress in skin tissue and has been discussed as an exogenous source of AGEs in the skin (Chen et al., 2022). Glycation stress occurs when a reducing sugar such as methylglyoxal, glyoxal, or glycolaldehyde combines with a protein; this is called the Maillard reaction, and is how AGEs are formed. In the skin, AGEs are involved in modifying skin collagens, which cause a decrease in skin elasticity that induces an increase in wrinkle formation (Masamitsu Ichihashi et al., 2011). UVB exposure increases the levels of carboxymethyl lysine, one of the major AGEs in elderly human skin, and accelerates skin aging by increasing oxidative stress and abnormally modified keratins (Mori et al., 2011). The AGEs act as ligands for AGE receptors, which are involved in skin diseases such as atopic dermatitis, lupus, leprosy, systemic sclerosis, diabetic skin, and ulcers. In addition, RAGE activation induces activation of the pro-inflammatory pathway and production of NF-κB, pro-inflammatory cytokines, tumor necrosis factor-α, and interleukin-1β (Guarneri et al., 2021). In this regard, treatment with anti-AGE substances could be a potential approach for protecting against UVB-induced skin photoaging. Phytochemicals from natural materials, including flavonoids, inhibit AGE formation, break AGEs, and block the AGE-RAGE axis (Shen et al., 2020). Furthermore, a previous study demonstrated the inhibitory effect of ST extract on AGE formation and AGE-protein crosslinking, and the results indicated that ST extract may be a potential substance for reducing AGE-related glucotoxicity in kidney cells (Do et al., 2019). In our study, ST ethanolic extract decreased AGE accumulation and RAGE expression in mice exposed to UVB-induced photoaging. These results are similar to those of other studies, where AGEs accumulated in the skin ECM structures and were highly expressed in the skin during UV-induced photoaging (Mizutari et al., 1997; Lohwasser et al., 2006). Our results suggest that ST ethanolic extract suppresses AGE formation, and its effect alleviates UVB-induced photoaging in HR-1 mice by downregulating RAGE.

In this study, we investigated the mechanisms underlying ST ethanolic extract’s protective effects against UVB-induced photoaging in HR-1 mice. Exposure to UVB causes skin damage, wrinkle formation, skin thickness, and skin dehydration, which were relieved by ST ethanolic extract treatment via regulation of skin wrinkle- and hydration-regulated proteins. Furthermore, ST ethanolic extract inhibited the expression of MAPKs and NF-κB. Additionally, ST ethanolic extract not only suppressed the accumulation of AGEs in skin tissue but also decreased the expression of RAGE. Taken together, these results suggest that ST ethanolic extract may be a potential candidate for anti-photoaging caused by UVB.

## Data Availability

The original contributions presented in the study are included in the article/Supplementary Material, further inquiries can be directed to the corresponding author.

## Abbreviations

AGEs, advanced glycation end products; ALT, alanine aminotransferase; AP-1, activating protein-1; AST, aspartate aminotransferase; ECM, extracellular matrix; ERK, extracellular signal-regulated kinase; HA, hyaluronan; HAS, hyaluronan synthase; H&E, hematoxylin and eosin; HR-1, HR-1 hairless mice; HYAL, hyaluronidase; IHC, immunohistochemistry; JNK, Jun N-terminal kinase; MAPK, mitogen-activated protein kinase; MMPs, matrix metalloproteinases; NF-κB, nuclear factor-kappa B; RAGE, receptor for AGEs; ROS, reactive oxygen species; ST, Schizonepeta tenuifolia; TBST, Tris-buffered saline containing 0.2% Tween-20; TIMP1, tissue inhibitor of metalloproteinase 1; UV, ultraviolet; UVB, ultraviolet B.
